# Enhanced Plasmonic Wavelength Selective Infrared Emission Combined with Microheater

**DOI:** 10.3390/ma10091085

**Published:** 2017-09-14

**Authors:** Hiroki Ishihara, Katsuya Masuno, Makoto Ishii, Shinya Kumagai, Minoru Sasaki

**Affiliations:** 1 Yazaki Corporation, Shizuoka 4101194, Japan; hiroki.ishihara@jp.yazaki.com (H.I.); katsuya.masuno@jp.yazaki.com (K.M.); makoto.ishii@jp.yazaki.com (M.I.); 2 Toyota Technical Institute, Aichi 4688511, Japan; kumagai.shinya@toyota-ti.ac.jp

**Keywords:** wavelength selective infrared emitter, indirect plasmonic thermal emitter, surface plasmon polariton, thermal isolation, microheater

## Abstract

The indirect wavelength selective thermal emitter that we have proposed is constructed using a new microheater, demonstrating the enhancement of the emission peak generated by the surface plasmon polariton. The thermal isolation is improved using a 2 μm-thick Si membrane having 3.6 and 5.4 mm outer diameter. The emission at around the wavelength of the absorption band of CO_2_ gas is enhanced. The absorption signal increases, confirming the suitability for gas sensing. Against input power, the intensity at the peak wavelength shows a steeper increasing ratio than the background intensity. The microheater with higher thermal isolation gives larger peak intensity and its increasing ratio against the input power.

## 1. Introduction

The blackbody is still used as the mid-infrared (IR) emitter having a broad spectrum. Since the mid-IR gives the information of the molecular vibration which can identify the molecule, there are many applications, especially relating with the gas sensors (e.g., air pollution monitoring, fire alarms, noninvasive medical sensing). When the molecular vibration changes its dipole moments, absorption peaks in the IR region will be observed. Many gases show their own IR absorption peaks. CO_2_ is one of the common gases in air. CO_2_ concentration is regarded as one of the important factors of indoor air quality. Based on the chemical or physical characteristics of the gas molecules, there are many sensing methods. For CO_2_ gas sensing, methods based on chemical reaction are not appropriate, since CO_2_ gas has little chemical activity, being the resultant gas after the combustion [1].

The typical compact gas sensor is the non-dispersive IR (NDIR) sensor [2]. This principle of this sensor is based on the fact that the molecules have intrinsic IR absorptions. For example, the absorption band of CO_2_ gas is at the wavelength of 4.2–4.3 μm. This is unique for each gas [3]. So, the cross-sensitivity against many gases can be avoided from the sensing principle. By changing the sensing wavelength, other gas molecules (e.g., CO: 4.5–4.8 μm, H_2_O: 2.6–2.7 μm) can also be measured. However, the low power efficiency is a weak point. The spectrum of the blackbody emitter is significantly broad compared to the bandwidth of the gas absorption (about 100 nm). The gas sensing only uses IR having a wavelength at the gas absorption band. In case of the broad spectrum having the wavelength outside that, the almost emitted IR is not used for the gas sensing. The semiconductor-type wavelength selective emitters for the mid-IR are still technically difficult. The fluctuation caused by the ambient temperature has a significant influence on narrow bandgap devices. Light-emitting diodes have low quantum efficiency with an operation temperature limit [4]. Quantum cascade lasers are in the market from some companies. However, they are too expensive to use in NDIR gas sensors. IR emitters having wavelength selectively near to the absorption bandwidth are required. The plasmonic thermal emitter is a promising candidate [5,6,7].

**Figure 1:**
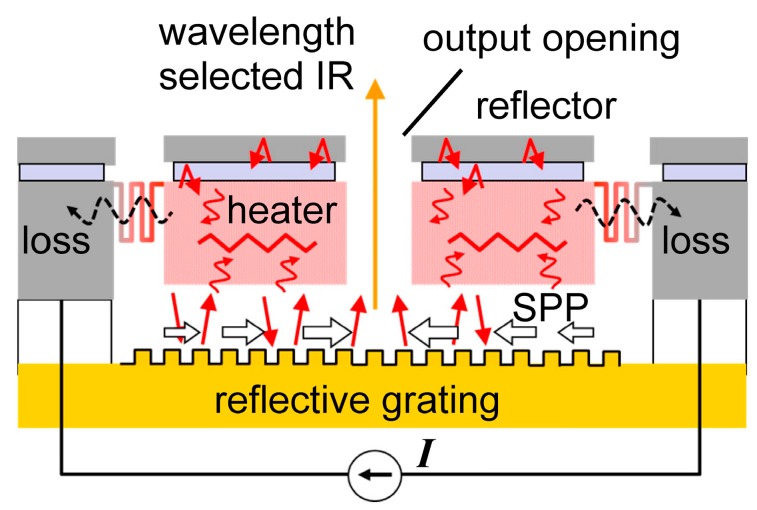
Schematic drawing of the indirect wavelength-selective thermal emitter and the energy flows.

Figure 1 illustrates the proposed emitter [8]. The heater emits IR based on the blackbody. However, this emission is fundamentally confined in the cavity constructed by two reflectors sandwiching the heater. The exception is the propagation as the surface plasmon polariton (SPP) on the grating. The SPP is excited beneath the heater carrying IR energy. IR emission is the reverse process of SPP excitation at the area open to the outside (center in Figure 1). According to Kirchhoff’s law of thermal radiation, the absorbance is equal to its emittance. Since the SPP excitation condition is narrow (wavelength, incident angle), wavelength selectivity is obtained. The 1/*e* propagation distance of SPP on Au surface is estimated to be 3.5 mm at the wavelength of 4.0 μm [9]. Such a long distance means that SPP propagation is efficient for carrying IR power in microdevices. Since the grating is separated from the heater, the grating can be set at low temperature. As for the heater, the grating structure is not necessary, and a wide variety of designs can be allowed. The requirement is that a higher temperature region with smaller power be generated to improve the power efficiency.

In this study, an indirect thermal emitter is constructed using a new microheater to obtain wavelength-selective IR emission with improved power efficiency, taking advantage of the higher thermal isolation of the membrane structure [10].

## 2. Device Concept

Figure 1 includes the energy flow inside the device. The total emission power from the blackbody is decided by only the temperature as *σT*^4^ (*σ* is constant) according to Stefan–Boltzmann law. So, the role of the microheater is to make a higher temperature region with lower power consumption [11,12]. Thermal conduction is the largest loss factor, and must be minimized first. The thin membrane structure is known to be useful for obtaining a higher temperature using lower power. This approach is used in un-cooled IR detectors [13,14], flow sensors [15], and acceleration sensors [16]. The high-temperature region in the mid-IR emitter tends to be larger compared to the active area in quantum devices like the laser diode. The blackbody IR emitter from Leister company has the membrane size of 2.1 × 1.8 mm^2^ [17]. The grating has the additional role of gathering SPP from the wider region to the center.

**Figure 2:**
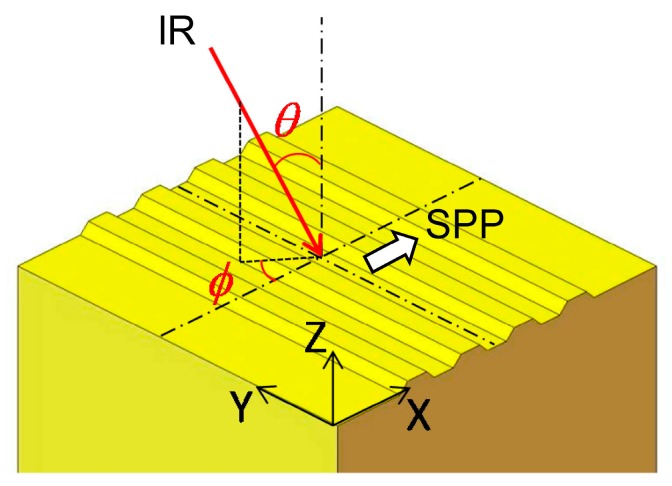
Schematic drawing of the incident light on the reflective grating. SPP: surface plasmon polariton.

Figure 2 shows the schematic drawing of the reflective grating placed beneath the microheater. The conservation of momentum requires the wavelength selection described by the following equation [18]:

(1) $$k_{sp}=k_{i}+mK=n_{d}k_{0}\sin\theta [\cos\varphi \hat{x}+\sin\varphi \hat{y}]+m\frac{2\pi }{\Lambda }\hat{x}(m=\pm 1,2,3\cdots )$$

where ***k****_sp_*, ***k****_i_*, and ***K*** are the wave vectors of SPP, the incident light, and the grating, respectively. *n_d_* is the refractive index of the dielectric medium above the grating, *k*_0_ is the magnitude of the wave vector of the light in free space, and *Λ* is the grating pitch. The incident angles are the polar angle *θ*, and the azimuthal angle *φ*. The grating used in this study is the same as the one used in the previous study [19], which showed the emission at around 4.3 μm using the Nichrome-wire heater. The grating pitch is 4.3 μm, and the groove depth is 0.25 μm. This shallow groove gives a relatively simple SPP excitation. The wavelength peaks distribute with the incident angle. The peak wavelength *λ* coupled with SPP starts from almost the same value with the grating pitch *Λ* [20]. According to Equation (1) with the larger polar angle *θ*, *k*_0_ = 2π*/λ* can be smaller. This means *λ* coupled with SPP becomes longer. Considering *x* direction, with the larger azimuthal angle *φ*, *λ* coupled with SPP becomes shorter. The proposed emitter will include such SPP-related emissions. The grating used consists of a gold film on the Si grating base. The shallow grating base is easy to fabricate. The underlying Si substrate has no effect, since the gold film is thicker than the skin depth of the field.

## 3. Design

**Figure 3:**
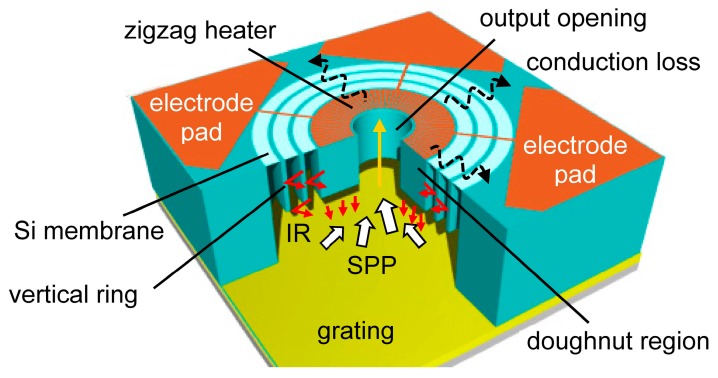
Schematic drawing of the microheater on the grating. There is alignment tolerance between the microheater and the underlying grating since the grating is uniform.

Figure 3 shows the schematic drawing of the emitter using a new microheater. The thin Si membrane decreases the thermal conduction loss from the higher-temperature center doughnut region to the lower-temperature substrate region. The structures are basically circular, and can decrease the stress focusing, even when the temperature increases. The zigzag heater is made of thin Cr film. IR emission passes through the center output opening after propagating on the grating as SPP. The vertical ring is for strengthening the membrane structure mechanically. Although this ring is the thick Si structure, the thermal conduction from the center to the surrounding substrate is suppressed, since this ring is self-closed not contacting to the outside. Cr has the relatively lo w thermal conduction among metals. The electrical connection from Cr electrode pad to the outside circuit is easier compared to the previous Si microheater device [21].

## 4. Fabrication

**Figure 4:**
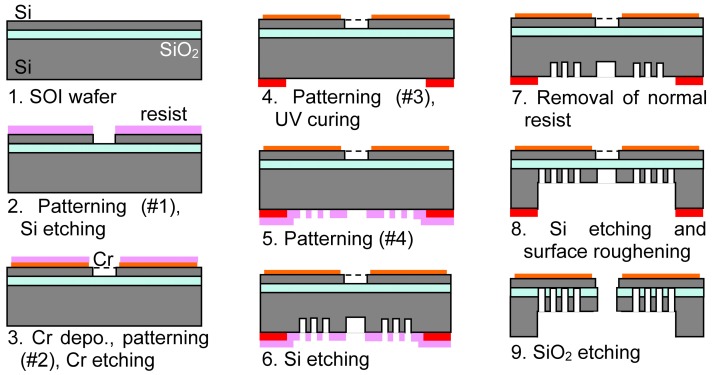
Fabrication sequence of the microheater with the thin membrane.

Figure 4 shows the fabrication sequence. (1) The Si-on-insulator (SOI) wafer is the starting material. The thicknesses of the device Si (*p*-type with the resistivity >1000 Ω-cm), the buried oxide, and the handle Si layers (*p*-type, 0.001–0.002 Ω-cm) are 2, 0.5, and 250 μm, respectively; (2) The center opening is patterned and opened using Si etching; (3) The Cr layer (about 100 nm thick) is sputter-deposited, and is patterned as the zigzag heater; (4) The backside is patterned and UV-cured defining the substrate base structure; (5) Since UV-cured resist shape becomes stable against the thinner and the developer, the additional resist film is over-coated and patterned, defining the doughnut and ring structures using the normal resist film; (6) The vertical Si etching is applied for about 125 μm; (7) The top normal photoresist is removed after the flush-exposure and the development, keeping the underlying UV-cured resist pattern; (8) The vertical Si etching reaches the buried oxide; (9) The appeared buried oxide is etched using the buffered HF solution.

**Figure 5:**
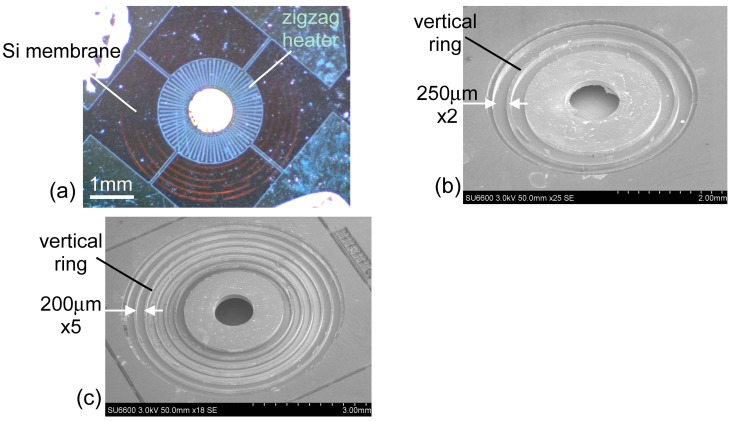
(**a**) Reflection photo of the microheater. The heating current flows between two electrodes at the opposite corners. The upper electrode pads have a silver paste for connecting with the outside circuit; (**b**,**c**) SEM images of the microheaters observed from the backside. When the driving current flows, the membrane is observed to deflect vertically.

Figure 5a shows the reflection image of the microheater observed from the front side. The Si membrane is shown in orange. Figure 5b,c are SEM images observed from the backside, having membrane outer diameters of 3.6 and 5.4 mm corresponding to the total ring widths of the thin membrane of 500 (2 times 250 μm-wide ring membrane) and 1000 μm (5 times 200 μm-wide ring membrane), respectively. The doughnut region and the vertical rings are 125–129 μm in height. The inner diameter of the output opening is 1 mm. The doughnut structure has a rough surface as a result of the Si etching. This roughness may decrease the reflection, increasing IR emission from the hot Si region.

## 5. Experiment

**Figure 6:**
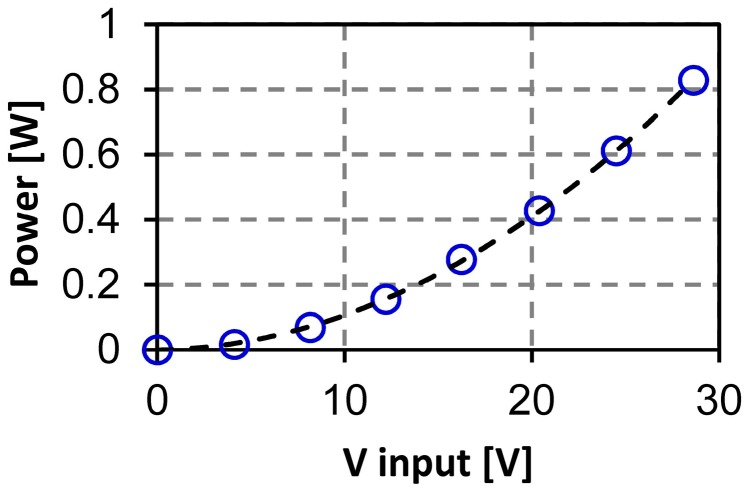
The consumed power as a function of the input voltage of one typical device.

Figure 6 shows the typical curve of the consumed power as a function of the input voltage. With the voltage, the power increases nearly following a parabolic relation. The resistance between the electrodes is about 1 kΩ. The operation is maintained within 35 V. At higher voltage, this resistance tends to decrease and sometimes breaks mechanically. The Si membrane may become conductive due to the thermally excited carriers over the bandgap [22].

**Figure 7:**
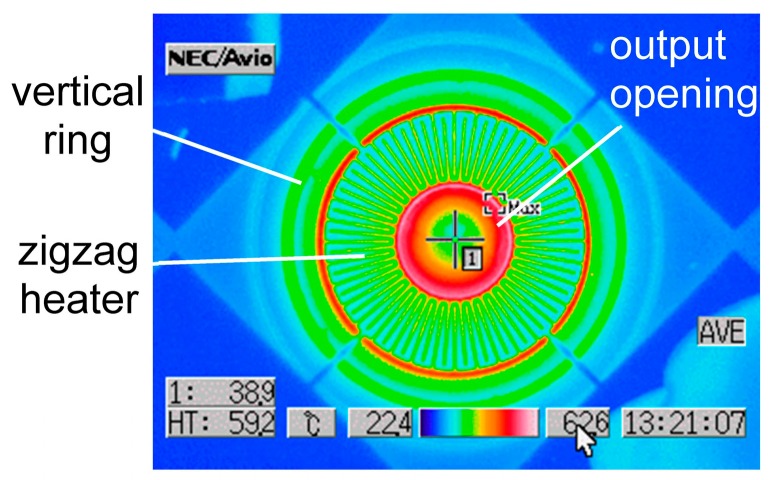
Thermography image of one microheater driven at 17 mW (15 V, 1.13 mA). A NEC Avio TVS-500 EX with microscope lens was used.

Figure 7 shows the thermography image of the emitter driven in air at the input power of 17 mW. This microheater is stacked on the gold grating. In general, the higher temperature region emits larger IR intensity. The larger IR emission is observed inside the output opening. This region corresponds to the underlying grating having the lower room-temperature, at which the blackbody emission should be small. So, the emission from the center opening will relate to SPP propagated on the grating. The color gradation from the opening peripheral to the center will indicate the density distribution of SPP. The zigzag heater covers the almost high temperature doughnut region, and its high reflectivity suppresses IR emission, being observed as the lower temperature. The red ring outside the zigzag heater corresponds to the Si region without Cr cover. So, the emission from the center opening is mixed with the blackbody emission at present. The substrate is almost at room-temperature. The temperature distribution on the Si membrane indicates effective thermal isolation. The temperature changes stepwise at the ring. The vertical ring seems to reflect IR back to the center, confining the thermal energy.

**Figure 8:**
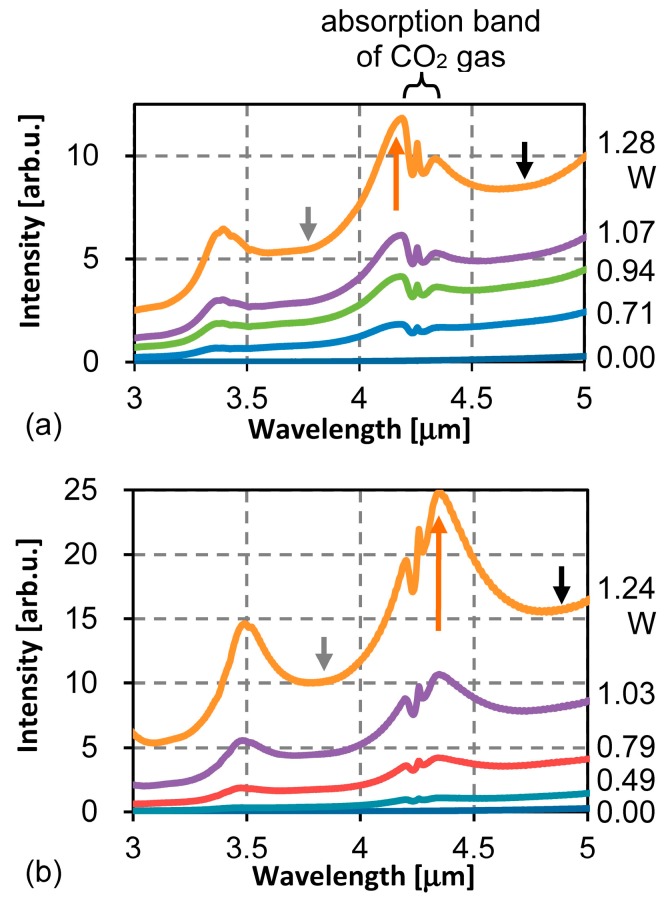
Fourier transform infrared (FT-IR) spectra of the emission. The total membrane ring widths are (**a**) 500 μm and (**b**) 1000 μm. Arrows indicate the wavelengths plotted in Figure 9.

Figure 8a,b show FT-IR spectra of the emission obtained using the microheaters shown in Figure 5b,c, respectively. At present, the shape of the spectra is different from device to device because the profile of the gold grating used is not perfectly identical. The IR output intensity of Figure 8b is nearly double that of Figure 8a. This is consistent with the fact that the total ring width of the thin membrane is doubled. The emission peaks increase with the input power. The peak at around 4.3 μm has the width of about 400 nm, inside which the CO_2_ absorption band is observed. The absorption signal also grows in the increased emission peak profile. 

**Figure 9:**
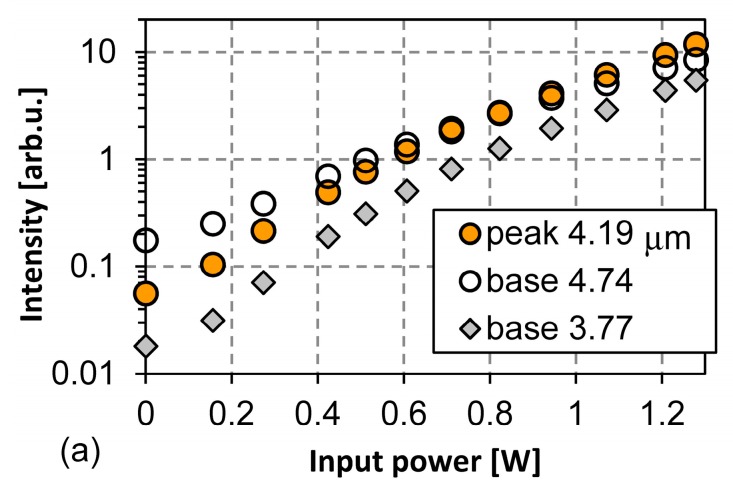

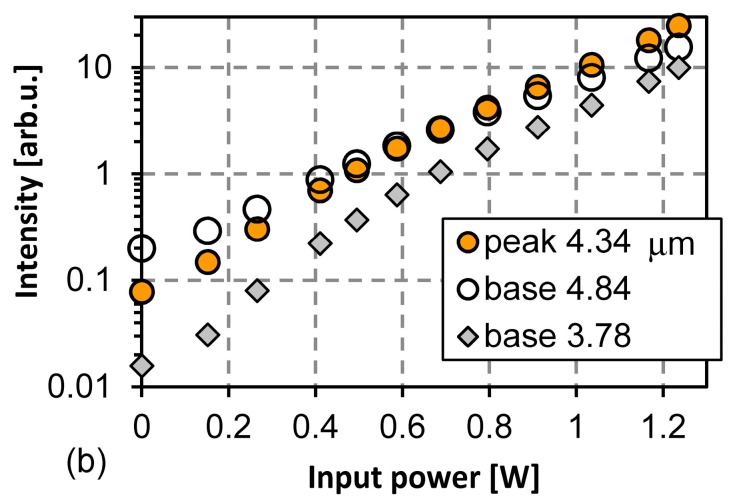
IR intensity as a function of the input power. The total membrane ring widths are (**a**) 500 μm and (**b**) 1000 μm. The vacuum package will decrease the loss and increase the intensity further.

Figure 9a,b plot IR intensity as a function of the input power corresponding to Figure 8a,b. Three wavelengths correspond to the peak wavelength and the shorter and the longer wavelengths of the background base which correspond to the blackbody emission, respectively. Against the input power, the intensity at the peak wavelength increases more steeply compared to the background intensity. This tendency continues from the beginning to the maximum examined power of about 1.2 W. At around 0.7 W, the peak intensity exceeds the intensity of the longer wavelength of the background base. This cannot be explained by the simple wavelength filtering of the blackbody emission. This shows the wavelength-selective emission.

## 6. Discussion

Figure 8 includes the peaks at 2.9 and 3.5 μm with the designed peak at around 4.3 μm. Such peaks are also reported in the previous studies [7,8,19]. The theoretically calculated absorption spectrum for exciting SPP at a single condition has a peak width of about 10 nm. Figure 8 shows a much wider peak. The observed emission peak is the sum of the theoretical peaks at many conditions. As explained using Equation (1), the peak wavelength changes when the incident angle changes. Since the emitter setup allows the wide conditions of the incident angle (polar and azimuthal angles) on the gold grating and the polarization direction, the peaks make groups giving the resultant emission peaks. Additionally, there are fluctuations from device to device, as shown in Figure 8a,b. This can be attributed to fluctuations of the grating profile at present. The characteristics of the grating for absorbing the specific IR as SPP is mainly decided by the grating pitch, the groove depth, and the duty ratio of the groove. Although the grating pitch is maintained during the fabrication, the groove depth and the duty ratio can change. The etching ratio of Si and the deposition ratio of Au are not perfectly uniform, having position dependence.

The intensity curves shown in Figure 9a,b seem to have two parts having two different increasing ratios. The changing points are around 0.7 W. This is the power when the peak intensity crosses with the intensity of the longer wavelength of the background base. The major factor of the emission may change. The approximate equations for the peak intensity in Figure 9a at the regions below 0.6 W and over 0.7 W are proportional to *e*^5.24*P*^ and *e*^3.26*P*^, where *P*[W] is the input power. Additionally, those in Figure 9b are *e*^5.42*P*^ and *e*^4.00*P*^, respectively. The increasing ratios in Figure 9b are larger than those in Figure 9a. This indicates that the thermal efficiency of the microheater strongly relates with the IR emission performance. By introducing a microheater which can reduce thermal loss with better thermal isolation, the thermal energy will be well directed to wavelength-selective emission. 

## 7. Conclusions

A new microheater is developed for an indirect wavelength selective thermal emitter with a center opening and improved thermal isolation. The mm-size 2 μm-thick membrane is realized combined with a ring structure. The emission spectrum has clear peaks which cannot be explained by simple blackbody emission. The peak wavelength does not change when the input power increases. The peak intensity shows a steeper increasing ratio against the input power compared to the intensity at the surrounding base wavelength.
